# Beyond government accountability: the role of medical schools in addressing the NHS workforce crisis

**DOI:** 10.1177/01410768231209021

**Published:** 2023-10-31

**Authors:** Tomas Ferreira

**Affiliations:** Department of Clinical Neurosciences, School of Clinical Medicine, University of Cambridge, Cambridge, CB2 0PY, UK

Recently, we conducted the Ascertaining the Career Intentions of Medical Students (AIMS) study, the largest ever UK medical student study, investigating students’ career intentions post-graduation.^1,2^ This cross-sectional web-based survey, benefiting from a network of approximately 200 collaborators, revealed that over one-third of medical students intend to abandon the National Health Service (NHS) within 2 years of graduating, either to practise abroad or to leave the profession entirely. Factors steering these decisions include dissatisfaction with remuneration, poor working conditions of a doctor in the NHS and an undesirable work–life balance.^1^ Similarly, reasons for medical students’ leaving the NHS are consistent with previous studies carried out on doctors.^3–8^ Furthermore, the study reported that only 17% of medical students are satisfied with the overall prospect of working in the NHS.^1^

While responsibility of systemic change to address these trends lies with the government, these findings raise questions regarding what role medical schools may assume in reversing these trends. Addressing this question demands an in-depth understanding of the underlying issues combined with the development of strategic initiatives aimed at retaining future doctors in the UK’s healthcare system. This commentary seeks to explore the potential avenues that can be developed to mitigate the emerging challenges associated with the NHS workforce crisis.

## Collaboration and student engagement

Countries such as Australia and New Zealand have successfully implemented annual census-style surveys to examine the career intentions of medical students, facilitating a proactive, data-informed approach to workforce planning, policy development and implementation.^3^ In the UK, it has taken a student-led initiative – the AIMS study – to bring to light important data concerning the future workforce of the NHS.^1^ This approach has its limitations, underscoring the necessity for the establishment of a regularised system of career intention assessment, which aligns with government and industry efforts in workforce planning and the improvement of doctor and student satisfaction levels. Furthermore, while the AIMS study has paved the way for longitudinal research, having secured consent for follow-up studies, a government-backed initiative could potentially provide a more comprehensive dataset.^9^ By mandating these studies, the government can secure a consistent stream of in-depth insights, equipping policy makers with the necessary information to implement policies that are both informed and predictive of future trends. Such an approach would signify a shift from traditional reactive measures to dynamic policy formation, constantly evolving in response to fluctuating medical students' aspirations and concerns and setting the stage for a healthcare system that is robust, adaptive and aligned with the needs and goals of its future workforce.

Despite being the largest survey of its kind in the UK, the AIMS study encountered resistance from select medical schools and the wider regulatory bodies, particularly in facilitating broader dissemination to increase the reproducibility of findings. Medical schools should foster a collaborative spirit, continuously monitoring student sentiments and career intentions, and encouraging student participation in health policy discourse, rather than stifling it. This approach advocates for a feedback-loop between medical schools and the government, fostering policy influences based on real-time data and insights.

Moreover, it is necessary to initiate an industry-wide dialogue encompassing viewpoints of current students. A possible avenue could be the creation of a national medical student advisory committee. This committee, comprising student representatives from various years and schools, would provide insights into the educational experience and act as an important stakeholder in discussions prior to the establishment of policies affecting medical schools or students. To safeguard its effectiveness and integrity, measures must be instituted to deter the involvement of individuals solely pursuing career advancement, thus encouraging a membership genuinely committed to improving health policy and medical education.

## Medical school admission processes

In addressing the findings of the recent AIMS study, a potential area for improvement lies in the admission procedures to medical schools. Currently, prospective students undergo varying forms of interviews, which, due to their brevity and the substantial volume of applications, may not adequately capture a candidate's realistic expectations and motivations towards a medical career. To increase the robustness of the selection process, medical schools should consider revisiting the structure of their interview processes, potentially incorporating methods to assess applicants' understanding and enthusiasm for a medical career within the NHS more accurately. This approach could include comprehensive discussions focusing on the complexities and realities associated with a medical career. Despite current working conditions instigating attrition, medical school admission policies should evaluate candidates’ understanding of the NHS and their dedication and preparedness for a medical career.

## Reassessing the expansion of medical school places

The recent NHS Long Term Workforce plan, proposing to double the number of medical school places, raises several questions.^10,11^ First, there is a concern over the availability of trainers for the increasing number of students, given the current – and growing – scarcity of medical professionals. Moreover, educational infrastructure is already strained with limited spaces for students in wards, theatres and clinics, potentially diluting the quality of education further.^10^ The anticipated expansion is likely to exacerbate existing bottlenecks at various stages, from the Foundation Programme^12^ to specialty posts,^13^ to consultant posts,^14,15^ making it critical to reassess the viability of this expansion without addressing underlying issues. Failure to do so may intensify the workforce crisis, potentially leading to a surge of doctors who, unable to further specialise, feel disillusioned and opt to leave, further straining resources.

Medical schools are integral in this discussion. They have a duty to advocate for both current and future medical students, resisting any changes that risk diluting the quality of education or harming the career prospects of their students through increased bottlenecks. The 'leaky bucket' analogy succinctly illustrates why increasing the number of medical students without rectifying underlying problems could be a misguided use of resources. As indicated by the AIMS study, if students intend to leave the NHS, as many doctors do, then merely increasing medical school places is an ineffective response.

Before expanding medical school places, a comprehensive plan must be devised in coordination with relevant stakeholders. Expansion plans should be phased in nature, where the increase in student intake is proportionate to the increase of necessary resources, thereby preserving the quality of education. Studies should also be commissioned to evaluate the potential impact of increasing the number of medical school places on the quality of education and the subsequent effects on the NHS workforce. These studies should be ongoing, conducted before, during and after the expansion to monitor the effectiveness of the changes continuously. If the results indicate a decline in education quality, provisions should be established to amend or retract the expansion to protect the integrity of medical education and the future NHS workforce.

## Curricular changes

Medical schools should consider revising their curricula to incorporate modules that provide insights into the NHS's structure, functions and current challenges. This could include discussions on healthcare policies, workforce management, potential career paths into health politics and the socioeconomic aspects of healthcare delivery in the UK. A curriculum grounded in the realities of the NHS could potentially nurture students who are not only aware but prepared to actively contribute towards improving the system.

Furthermore, while it is critical to prepare students to navigate the inherent challenges of medicine, presently, medical schools potentially foster a survivalist mentality that overlooks systemic issues.^16^ Medical schools must foster a dialogue extending beyond urging future doctors to develop resilience as a means to endure a flawed system. There is a need to shift the narrative from merely building resilience to poor working conditions to fostering an environment wherein doctors can thrive, both professionally and personally. Medical schools should not portray medicine as a hardship to endure. Instead, the objective should be to instigate systemic changes that foster a mindset encouraging growth and wellbeing, rather than survival.

## Career development and mentorship

It is important that medical schools cultivate a sense of clinical preparedness and professionalism from the early, non-clinical years of the programme. While implementing this in initial years may pose a challenge, considering the academic demands on students, it is indispensable for fostering a generation of clinicians rooted in realism and passion for their future roles. A potential strategy could involve introducing compulsory sessions that provide a glimpse into a day in the life of various specialties, encouraging students to envisage a tangible and promising future within the health system. However, care must be taken to avoid overwhelming students or pressuring them into premature career decisions.

In parallel, the integration of workshops focusing on curriculum vitae development and career guidance into the programme would provide students with a comprehensive view of their potential career trajectories. Currently, these initiatives are often coordinated by student-led societies, which, despite their efforts, might not reach a wide audience due to voluntary participation. By integrating these elements formally into the curriculum, medical schools can potentially foster a more informed, resilient and dedicated cohort of future NHS clinicians, thus aiding in reducing attrition.

Medical schools could try implementing more robust mentorship programmes where experienced doctors guide medical students, offering realistic expectations, which could prevent disillusionment. These programmes, with regular periodic encounters, could foster a connection with a prospective future in the NHS, helping students to envisage a career path and thereby increasing retention through engagement. Mentors can also guide students on how to maintain a healthy work–life balance, an issue raised by both doctors and medical students intending to leave the NHS.^1,3–8^

## Conclusion

The AIMS study highlights a significant trend of UK medical students considering paths outside the NHS post-graduation. While the government holds the primary responsibility for systemic change, medical schools may assist in reversing these trends. Medical schools are required to re-evaluate current curricula, admission processes and expansion strategies. Increased collaboration with government entities and fostering student engagement can potentially lead to the development of policies that are more aligned with the evolving needs and concerns of medical students. By adopting a data-driven approach to policy formation and fostering robust mentorship programmes, medical schools can contribute to stabilising the NHS workforce, ensuring a sustainable future for the UK's healthcare system.

## Declarations

### Provenance

Not commissioned; editorial review.

### Guarantor

TF.
